# Cognitive variability in healthy and impaired older adults: What is it? What causes it? How to measure it?

**DOI:** 10.1002/alz70857_101438

**Published:** 2025-12-25

**Authors:** Andrew J. Aschenbrenner, Muchen Xi, Ganesh M. Babulal, Jason J. Hassenstab, Joshua J. Jackson

**Affiliations:** ^1^ Washington University School of Medicine in St. Louis, St. Louis, MO, USA; ^2^ Washington University in St. Louis, St. Louis, MO, USA; ^3^ Washington University School of Medicine, Saint Louis, MO, USA; ^4^ Washington University School of Medicine, St. Louis, MO, USA

## Abstract

**Background:**

Cognition is not static but rather is subject to substantial variation across repeated assessments. The magnitude of “cognitive variability” can be measured with brief and high‐frequent cognitive assessments (HFCA). Despite the predictive utility of cognitive variability, many questions remain regarding its measurement and overall clinical utility. Specifically, does variability reflect a cognitive process or is it due to entirely task or situational‐specific influences? Which cognitive tasks are most amenable to high‐frequency administration and are most sensitive to outcomes of interest? What are the practical, real‐world implications of high cognitive variability? This presentation aims to answer these essential questions and provide a suite of ready‐made cognitive tasks suitable for an HFCA design.

**Method:**

We present data from three HFCA studies conducted at Washington University: the Ambulatory Research in Cognition (ARC) study (*N*∼300), the Cognitive Dynamics Study (*N*∼150), and the Cognitive Variability Battery (CVB, *N* ∼150), and an additional study, COGITO (*N* = 203). These data are used to examine whether cognitive variability clusters into distinct cognitive domains and determine which cognitive tasks correlate most strongly with daily predictors (e.g., stress). ARC is paired with a longitudinal driving study (DRIVES, *N* = 160) to determine if cognitive variability has functional implications for safety captured via naturalistic driving behavior.

**Result:**

Variability in tasks of episodic memory and processing speed were moderately to highly correlated (rs 0.40 – 0.86), but working memory variability was not (rs < 0.08), suggesting that working memory variability is highly situational. Only episodic memory variability was correlated with general intelligence. Additional metrics, including test reliability, will also be discussed. Poor daily cognitive performance was associated with a small but significant increase in adverse driving events (IRR = 1.06, *p* =  0.002).

**Conclusion:**

Cognitive variability clusters into standard cognitive domains, which implies that cognitive variability reflects true variability in cognitive processes rather than results from transient, task‐specific influences from the environment (e.g., momentary distractions). Moreover, specific deviations from mean performance have meaningful consequences for real‐world behavior, specifically driving. The CVB provides a ready‐to‐use suite of cognitive tests that are ideally suited to studying variability in daily life.

